# Accidents—Popular Directions for Their Immediate Treatment; with Observations on Poisons and Their Antidotes

**Published:** 1845-05

**Authors:** 


Accidents—*Popular directions for their immediate treatment; with Observations on Poisons and their Antidotes.* By Henry Wheaton Rivers, M. D., Surgeon to the United States Marine Hospital, Providence, R. I. 12mo. pp. 108. Thomas H. Webb & Co. Boston, 1845.

As a general rule, we look upon all attempts to popularize medicine by the publication of works containing directions for the treatment of accidents and diseases by those who have not devoted themselves to a thorough understanding of the science, as pernicious to the public, and disreputable to their authors. We are almost inclined, however, to make an exception in regard to the little work of Dr. Rivers. It is a plain, sensible, and unpretending production, better calculated for the object proposed by its author than any other of the kind with which we are acquainted. Young physicians will find it convenient for reference in the absence of more elaborate treatises, while intelligent laymen can hardly go wrong in following its directions in emergencies, when a skilful practitioner is not at hand.

